# Changes in gene expression associated with retinal degeneration in the *rd3* mouse

**Published:** 2013-05-06

**Authors:** Christiana L. Cheng, Robert S. Molday

**Affiliations:** Department of Biochemistry and Molecular Biology, Centre for Macular Research University of British Columbia Vancouver, B.C. V6T 1Z3 Canada

## Abstract

**Purpose:**

To identify and characterize changes in gene expression associated with photoreceptor degeneration in the *rd3* mouse model of Leber congenital amaurosis (LCA) type 12.

**Methods:**

Global genome expression profiling using microarray technology was performed on total RNA extracts from *rd3* and wild-type control mouse retinas at postnatal day 21. Quantitative PCR analysis of selected transcripts was performed to validate the microarray results.

**Results:**

Functional annotation of differentially regulated genes in the *rd3* mouse defined key canonical pathways, including phototransduction, glycerophospholipid metabolism, tumor necrosis factor receptor 1 signaling, and endothelin signaling. Overall, 1,140 of approximately 55,800 transcripts were differentially represented. In particular, a large percentage of the upregulated transcripts encode proteins involved in the immune response; whereas the downregulated transcripts encode proteins involved in phototransduction and lipid metabolism.

**Conclusions:**

This analysis has elucidated several candidate genes and pathways, thus providing insight into the pathogenic mechanisms underlying the photoreceptor degeneration in the *rd3* mouse retina and indicating directions for future studies.

## Introduction

Inherited photoreceptor degenerations are a diverse group of genetic disorders affecting many aspects of photoreceptor function and share the same ultimate outcome of photoreceptor cell death. The cellular and molecular pathways leading from the genetic mutation to the photoreceptor death are not well understood. Recently, research in naturally occurring and experimentally generated mouse models for photoreceptor degeneration using microarray technology revealed the coordinated expression of a similar set of genes during degeneration, suggesting that diverse genetic mutations converge onto parallel pathogenetic mechanisms [1]. The general genomic responses observed are the upregulation of transcripts associated with apoptosis [2-6] and immune-related processes [4,6] including the complement cascade [2,7] and the glial cell activation [2,3,8,9].

Leber congenital amaurosis (LCA) is one of the most severe inherited retinal degenerative diseases that cause blindness at birth or within the first year of life [10]. Mutations in the *RD3* gene cause LCA12 in humans [11,12]. Naturally occurring *Rd3* mutations in collie dogs and mice mimic the human LCA phenotype [11,13], and these animals serve as useful models for studying LCA12. Three strains of mice (RBF/DnJ, Rb(11.13)4Bnr, and In(5)30Rk) with the *Rd3* mutation have been identified and shown to have different rates of retinal degeneration [14,15]. The mouse *Rd3* mutation is a cysteine to thymidine substitution in exon 3 resulting in a stop codon after amino acid 106 and creating an unstable truncated RD3 protein [11]. One of the human *RD3* mutations is similar in that it results in a truncated protein of 99 amino acids due to mutation of a guanine to adenine at the end of exon 2 donor splice site [11].

The *Rd3* gene encodes a 195-amino acid long protein that is highly expressed in the retina and more specifically photoreceptor cells, where the protein binds to guanylate cyclase (GC) 1 and 2 (GC1 and GC2) as revealed by coimmunoprecipitation [16]. This transient interaction is part of a mechanism to translocate GCs from the ER to the photoreceptor outer segment and suppress the basal enzymatic activity of GCs [16,17]. GC1 and GC2 play an indispensable role in phototransduction by catalyzing the synthesis of the second messenger, cyclic guanosine monophosphate (cGMP), in photoreceptors [18]. In fact, GC1 was the first gene to be associated with LCA [19]. Interestingly, *rd3* mice lack GC expression in the retina, highlighting the importance of RD3 in maintaining GC expression and stability, in addition to regulating GC activity [16]. The fact that RD3 regulates multiple aspects of GCs points to RD3’s indirect significant contribution to phototransduction and photoreceptor cell viability. In agreement with this, the retinas of *rd3* mice exhibit a gradual extinction of electroretinography (ERG) coinciding with the time course of photoreceptor loss that starts at post-natal week 3 and is completed by 8–16 weeks depending on the strain of *rd3* mice [14]. Photoreceptor differentiation proceeds normally up to post-natal week 2, but the outer segments of photoreceptors develop into shortened, disorganized structures [15].

Although studies have established the significance of RD3 in the function and survival of photoreceptors, the pathogenic mechanism underlying *rd3*-induced photoreceptor degeneration remains unknown. We used microarray technology to examine the global gene expression changes in the degenerating *rd3* mouse retina to identify genes and molecular pathways that are potentially involved in photoreceptor cell death associated with LCA12.

## Methods

### Animals

BALB/c and Rb(11.13)4Bnr/J (4Bnr) mice were purchased from Jackson Laboratories (Bar Harbor, ME). 4Bnr mice have a naturally occurring *rd3* mutation that gives rise to photoreceptor degeneration starting around 3 weeks of age. This strain of mice also has a Robertsonian translocation between chromosome 11 and 13, which may or may not contribute to the severity of degeneration. The aim of our study was to examine the genetic changes associated with *rd3*-induced photoreceptor degeneration, and therefore, it was important to keep the genetic background constant between the experimental and the control mice. The appropriate control for the microarray experiment is the 4Bnr mouse with a wild-type (WT) copy of *rd3*. This strain is generated by crossing 4Bnr mice with BALB/c to obtain *rd3* heterozygotes, which were then interbred to obtain litters with potential *rd3* homozygotes (4Bnr-BALB/c-*Rd3^rd3/rd3^* as experimental) and WT (4Bnr-BALB/c-*Rd3^+/+^* as control). All mice were maintained under a 12 h:12 h light-dark cycle. Animals were treated in accordance with the Association for Research in Vision and Ophthalmology Statement for the Use of Animals in Ophthalmic and Vision Research. All procedures and protocols conformed to the University of British Columbia (UBC) policies and were approved by the UBC Committee on Animal Care.

The *rd3* mutation was genotyped using the following primers: forward- 5′ CAA GAG CAA GGT TGG GAG TT 3′; reverse- 5′ TCC AGC ATT CAA GGA CTC AG 3′. PCR was performed with mouse ear DNA extracted with the REDExtract-N-Amp Tissue PCR Kit (Sigma, St. Louis, MO) using standard conditions. Amplified products were sequenced at Genewiz (Seattle, WA).

### Microarray

Mice at postnatal day (P) 21 were sacrificed and the retinas isolated and stored in RNA-later (Ambion) at −30 °C. The following microarray procedure and data analysis were performed by the Laboratory for Advanced Genome Analysis at the Vancouver Prostate Centre (Vancouver, Canada). Total RNA was extracted from mouse retina samples (MirVana miRNA Isolation Kit, Ambion, Grand Island, NY), and the quality was assessed with the 2100 Bioanalyzer (Agilent Technologies, Santa Clara, CA). Samples with an RNA Integrity Number (RIN) greater than or equal to 8.0 were deemed acceptable and were labeled as probes for microarray analysis.

Cyanine-3-labeled cRNA probes were hybridized on Agilent SurePrint G3 Mouse GE 8×60K Microarrays (Design ID 028,005). Arrays were scanned with the Agilent DNA Microarray Scanner. The data were processed with Agilent Feature Extraction 10.10, and quantile normalized and analyzed in Agilent GeneSpring 11.5.1. The total data set included four replicates for both groups. Each had two retinas from the same animal.

To find significantly regulated genes, fold changes between the compared groups and p values gained from the Student *t* test between the same groups were calculated. The Welch *t* tests were performed on normalized data that had been log transformed, and the variances were not assumed to be equal between sample groups. Differentially expressed genes with a fold change cutoff of 2.0 and a p value cutoff of 0.05 were further analyzed in Ingenuity Pathway Analysis (IPA) software (Ingenuity Systems, Redwood City, CA) and Database for Annotation, Visualization and Integrated Discovery (DAVID, v6.7 [20,21]).

### Quantitative polymerase chain reaction

Quantitative PCR (qPCR) was performed to validate a subset of the microarray data. Total RNA was extracted from retinal tissue as previously described. cDNA was synthesized from 0.5 µg total RNA with reverse transcriptase (iScript; Bio-Rad, Mississauga, Canada). Quantitative PCR was then performed (iTaq Universal Probe Supermix; Bio-Rad) according to the manufacturer’s instructions. Reactions were performed in triplicate. Amplification of *Gapdh* was used for normalization while amplification of *Cnga1* was used as a control since this photoreceptor-specific gene yielded no expression change as detected with the microarray. PrimeTime assays (primers and probes) used in the qPCR reactions were purchased from Integrated DNA Technologies (San Diego, CA).

### Immunofluorescence and confocal microscopy

4Bnr-BALB/c-*Rd3^rd3/rd3^* and 4Bnr/BALB/c-*Rd3^+/+^* mouse retinas were used for immunofluorescence labeling studies to confirm some of the gene expression changes detected in the microarray results. Whole mouse eyecups were fixed with 4% paraformaldehyde in 0.1 M sodium phosphate buffer (PB, pH 7.4) for 2 h, rinsed, cryoprotected in 20% sucrose overnight, embedded in Tissue-Tek OCT (Fisher Scientific, Ottawa, Canada), and cut into 10 µm cryosections. Cryosections were permeabilized and blocked with 0.1 M PB containing 0.2% Triton X-100 and 10% normal goat serum for 20 min and labeled overnight with the primary antibody diluted in 0.1 M PB containing 0.1% TritonX-100 and 2.5% normal goat serum. Primary antibodies used in this study were anti-GC1 hybridoma fluid (8A5) and anti-glial fibrillary acidic protein (GFAP) polyclonal antibody (Sigma). Sections were labeled for 1 h with fluorescent-labeled secondary antibody (Alexa 488 or Alexa 594; Invitrogen, Grand Island, NY) and 4’,6-diamino-2-phenylindole dihydrochloride nuclear stain (Invitrogen). The stained sections were examined under a Zeiss LSM700 confocal microscope (Carl Zeiss, Oberkochen, Germany).

The thickness of the outer nuclear layer (ONL) of four 4Bnr-BALB/c-*Rd3^rd3/rd3^* and four 4Bnr/BALB/c-*Rd3^+/+^* was measured from the central retinal cryosections stained with 4’,6-diamino-2-phenylindole dihydrochloride nuclear stain (Invitrogen). Standard deviations of the measurement mean were calculated.

## Results

### Retinal degeneration in newly bred *rd3* mice

P21 was chosen for the time of microarray analysis because it has been documented that photoreceptor loss accompanied by reduced ERG activity is evident by 3 weeks of age in all three strains of *rd3* mice [11]. This indicates that photoreceptor degeneration is well under way and the changes in gene expression responsible for the degeneration should be prominent and detectable at this time.

**Figure 1:**
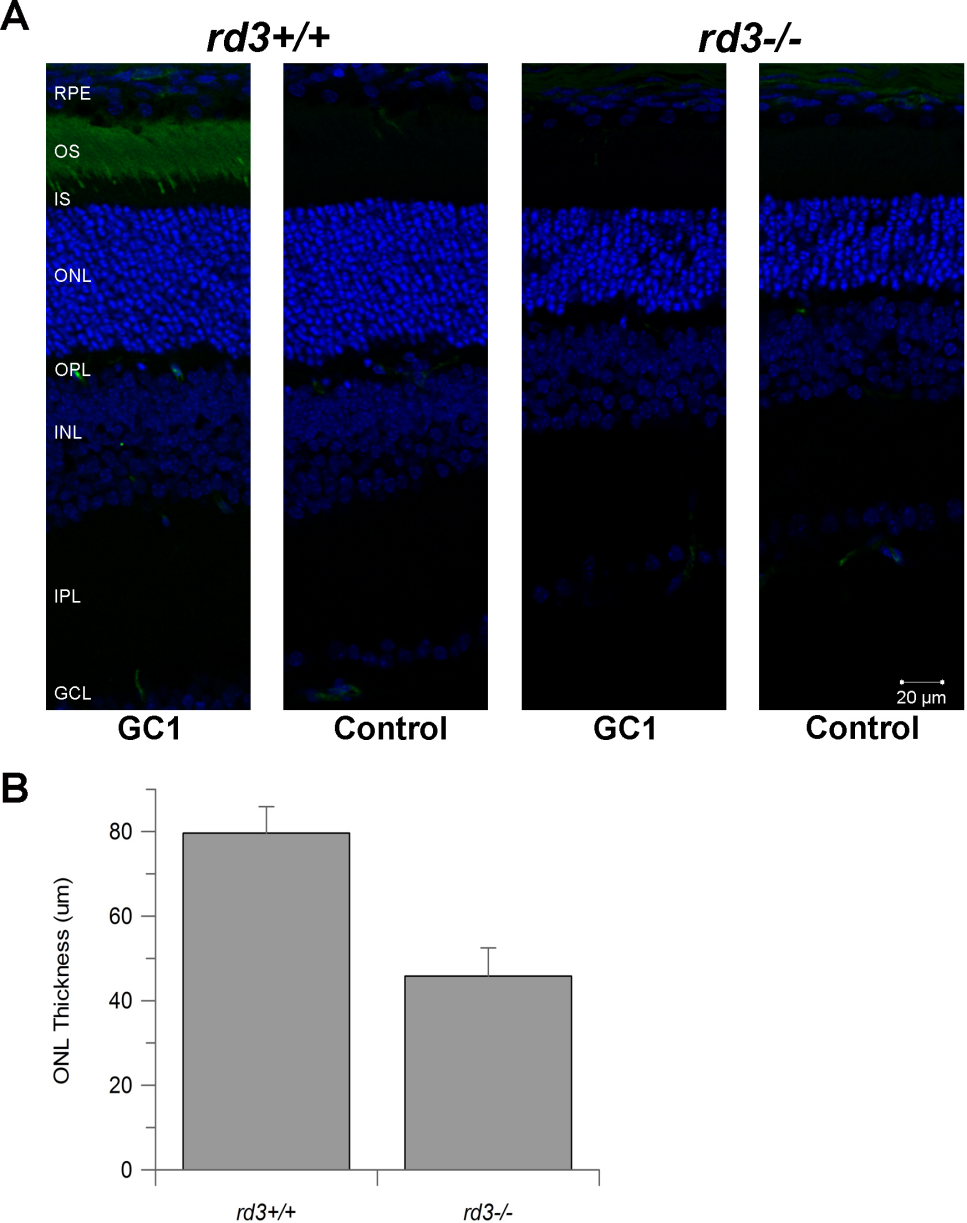
Comparison of the retina of 4Bnr-BALB/c-*Rd3^rd3/rd3^* (*rd3^−/−^*) and 4Bnr-BALB/c-*Rd3^+/+^* (*rd3^+/+^*) at P21. **A**: The nuclear layers stained with 4', 6-diamidino-2-phenylindole (blue) revealed that the outer nuclear layer (ONL) of *rd3^−/−^* is thinner than that of wild-type. Labeling with the antibody to GC1 (green) showed that GC1 is localized to the outer segments of the wild-type retina but is not detectable in the *rd3^−/−^* retina. In the control experiment where only the anti-mouse secondary antibody (Alexa 488) was used, punctate labeling in the OPL and the IPL was observed. The secondary antibody was likely labeling the retinal vasculature. **B**: The ONL thickness of *rd3^−/−^* was reduced by 42±4% compared to wild-type. Error bars represent the standard deviation of the mean (n=4). Abbreviations: RPE, retinal pigmented epithelium; OS, outer segment; IS, inner segment; ONL, outer nuclear layer; OPL, outer plexiform layer; INL, inner nuclear layer; IPL, inner plexiform layer; GCL, ganglion cell layer.

Similar to the other well-documented strains of *rd3* mice, loss of photoreceptors was observed in the newly bred 4Bnr-BALB/c-*Rd3^rd3/rd3^* mice at P21, as indicated by the thinning of the ONL (Figure 1A). The ONL thickness measured in the 4Bnr-BALB/c-*Rd3^rd3/rd3^* retina was reduced by 42±4% compared to that of 4Bnr-BALB/c-*Rd3^+/+^* mice (Figure 1B). The degree and rate of photoreceptor loss were comparable to those previously published where the reduction in the ONL was first detected at around 3 weeks of age [14,15]. The control used in this microarray study, 4Bnr-BALB/c-*Rd3^+/+^*, exhibits similar ONL thickness as age-matched wild-type BALB/c mice. In addition, this newly bred 4Bnr-BALB/c-*Rd3^rd3/rd3^* strain exhibits similar reduction in GC1 expression compared to Rb(11.13)/4Bnr (Figure 1A), proving that this strain is appropriate for studying the pathology underlying photoreceptor degeneration induced by *rd3* mutation.

### Microarray analysis

**Figure 2:**
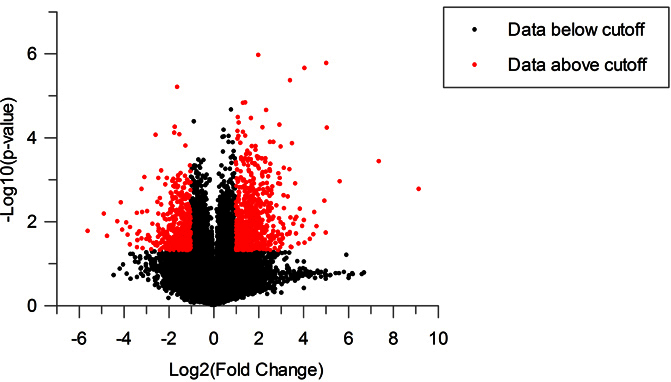
The proportion of significantly differentially expressed genes graphically presented in a volcano plot. Red points represent genes that pass the p<0.05 cutoff and have a fold change of greater than 2 (Log_2_=1).

To analyze gene expression changes associated with photoreceptor degeneration observed in the 3-week-old 4Bnr-BALB/c-*Rd3^rd3/rd3^* mice, Aligent microarray technology was used to identify differentially regulated genes. A total of 1,140 out of 55,820 genes, including annotated genes, uncharacterized genes, long intergenic non-coding RNAs (lincRNAs), expressed sequence tags (ESTs), and RIKEN cDNAs, were differentially expressed in the *rd3* retina when a twofold cutoff and p value <0.05 were applied (Figure 2). Of the differentially expressed genes, 812 are annotated genes, of which 507 are upregulated and 305 are downregulated (Appendix 1). The top ten genes differentially expressed in the *rd3* retina are listed in Table 1. Among the most upregulated genes are endothelin 2 (*Edn2*), glial fibrillary acidic protein (*Gfap*), and complement component factor I (*CfI*). Retina-specific genes are among the most downregulated, including phosducin (*Pdc*), gap junction protein α5 (*Gja5*), and retinal G protein-couple receptor (*Rgr*).

**Table 1:** Top 10 Most-Regulated Genes in the *rd3* Retina.

**Fold change**	**Gene symbol**	**Gene name**
**Top Upregulated Molecules:**		
165.2	*Edn2*	Endothelin 2
19.7	*Kars*	Lysyl-tRNA synthetase
13.0	*Chac1*	ChaC, cation transport regulator-like 1
12.8	*Ifi202b*	Interferon activated gene 202B
11.9	*Lad1*	Ladinin
11.4	*Gfap*	Glial fibrillary acidic protein
10.8	*Cfi*	Complement component factor i
10.6	*Ifit3*	Interferon-induced protein with tetratricopeptide repeats 3
8.9	*Nupr1*	Nuclear protein 1
8.8	*Btc*	Betacellulin, epidermal growth factor family member
		
**Top Downregulated Molecules:**		
6.8	*Foxp2*	Forkhead box P2
5.8	*Aknad1*	AKNA domain containing 1
4.8	*Acta1*	Actin, alpha 1, skeletal muscle
4.7	*Pdc*	Phosducin
4.7	*Ptgir*	Prostaglandin I receptor
4.4	*Gja5*	Gap junction protein, alpha 5
4.4	*Tnfaip3*	Tumor necrosis factor, alpha-induced protein 3
4.2	*Papolg*	Poly(A) polymerase gamma
4.2	*Rgr*	Retinal G protein coupled receptor
3.6	*Slco1c1*	Solute carrier organic anion transporter family, member 1c1

The most increased and decreased genes and their fold change are listed.

Thirteen of the differentiated genes are involved in human retinal disease (RetNet database), 11 showed significant increased level of expression, and two are significantly decreased in the *rd3* retina when compared to the control (Table 2). An additional six genes are associated with human disease loci [7]: Five are upregulated, and one is downregulated (Table 2).

**Table 2:** Differentially Expressed Genes in the *rd3* Retina involved in Human Retinal Diseases.

**Fold change**	**Gene symbol**	**Gene name**	**Retinal disease locus**
+4.4	*C3*	Complement component 3	ARMD9, ASP
−2.6	*Col9a1*	Collagen, type IX, alpha 1	
−2.5	*Crb1*	Crumbs homolog 1	LCA8, RP12
−2.3	*Dmd*	Dystrophin, muscular dystrophy	
−2.1	*Fam161a*	Family with sequence similarity 161, member A	RP28
−2.6	*Fscn2*	Fascin homolog 2, actin-bundling protein	RP30
−2.3	*Gnat1*	Guanine nucleotide binding protein, alpha transducing 1	CSNBAD3
−2.1	*Guca1b*	Guanylate cyclase activator 1B	RP48
+2.9	*Htra1*	HtrA serine peptidase 1	ARMD7, PRSS11
−2.2	*Opn1sw*	Opsin 1 (cone pigments), short-wave-sensitive	BCP, CBT
−2.6	*Pitpnm3*	PITPNM family member 3	CORD5, NIR1
−4.2	*Rgr*	Retinal G protein coupled receptor	RP44
−2.2	*Slc24a1*	Solute carrier family 24 (sodium/ potassium/calcium exchanger), member 1	CSNB1D, NCKX, RODX
			
**Genes assigned to human disease loci:**			
+7.6	*C1qa*	Complement component 1, q subcomponent, alpha polypeptide	LCA9, RP32
+4.9	*C1qb*	Complement component 1, q subcomponent, beta polypeptide	LCA9, RP32
+7.8	*C1qc*	Complement component 1, q subcomponent, C chain	NRL, RP27
+16.6	*Cebpd*	CCAAT/enhancer binding protein (C/EBP), delta (Cebpd)	CORD9
+2.3	*Egr1*	Early growth response 1	BSMD, PDE6A
−2.3	*Pla2g7*	Phospholipase A2, group	BCMAD, RDS, RP7

The associated disease locus of each gene is listed according to the RetNet database and Demos et al. [7].

**Figure 3:**
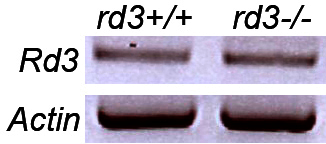
Transcript of *Rd3* is still present in the *rd3* retina. Reverse transcription–PCR using total RNA samples extracted from wild-type (*rd3^+/+^*) and *rd3^−/−^* retinas. Actin primers were used as the control.

Intriguingly, expression of the *Rd3* transcript in the *rd3* retina was not significantly reduced compared to the control retina. The presence of the *Rd3* transcript in the *rd3* retina was confirmed with reverse-transcription-polymerase chain reaction (Figure 3). This finding is not surprising because the naturally occurring mutation in the *rd3* allele presumably gives rise to an altered transcript that results in a premature truncation of the RD3 protein. Studies have detected the truncated RD3 protein by immunoblot analysis and by immunocytochemistry of COS-1-transfected cells [11], implying that the mutant *Rd3* mRNA is present in the retina of the *rd3* mice.

### Potential pathways responsible for photoreceptor degeneration

To gain insight into the differentially expressed genes in the *rd3* retina, two annotation tools (IPA and DAVID) were used. IPA identifies molecular networks and biologic pathways that are most perturbed in the data set of interest in a tissue-relevant context. The top ten canonical pathways in the *rd3* retina identified with IPA with a retinal-tissue filter are presented in Table 3. Of interest are the phototransduction pathway and the glycerophospholipid metabolism; the other pathways are associated with immunological responses, such as tumor necrosis factor receptor 1 signaling, acute phase response signaling, endothelin signaling, complement pathway, and Fcγ receptor-mediated phagocytosis in macrophages and monocytes.

**Table 3:** Top Canonical Pathways Affected in the *rd3* Retina.

**Top Canonical Pathways**	**Molecules**
Phototransduction	GNB3, GNAT1, PDC, OPN5, RGR, GUCA1B, GUCY2F, OPN1SW
Glycerophospholipid Metabolism	HMOX1, GPD1, CDS1, PNPLA3, AGPAT2, PLA2R1, ENPP2, PLCH2, PLA2G7
TNFR1 Signaling	JUN, PAK6, CRADD, TNFRSF1A, TNFAIP3, XIAP
Acute Phase Response Signaling	HMOX1, SERPING1, JUN, C3, TF, TNFRSF1A, CP, SERPINA3, OSMR, STAT3
Signaling by Rho Family GTPases	ROCK2, JUN, GNB3, GNAT1, PAK6, ARPC1B, VIM, GFAP, PARD3, ACTA1, MSN
Endothelin-1 Signaling	HMOX1, JUN, GNAT1, PNPLA3, PLA2R1, GUCY2F, PLCH2, PLA2G7, OPN1SW
Complement System	SERPING1, C3, CFI, C1QA
Phospholipid Degradation	HMOX1, PNPLA3, PLA2R1, ENPP2, PLCH2, PLA2G7
Fcγ Receptor-mediated Phagocytosis in Macrophages and Monocytes	HMOX1, RAC2, ARPC1B, HCK, FGR, ACTA1
Aryl Hydrocarbon Receptor Signaling	TGM2, NCOA7, JUN, CDKN1A, ALDH1L1, NCOA3, AHR

The list was generated with input of all genes that are significantly differentially expressed in the *rd3* retina. Pathways and the participating molecules as identified by Ingenuity Pathway Analysis (IPA) are listed.

**Figure 4:**
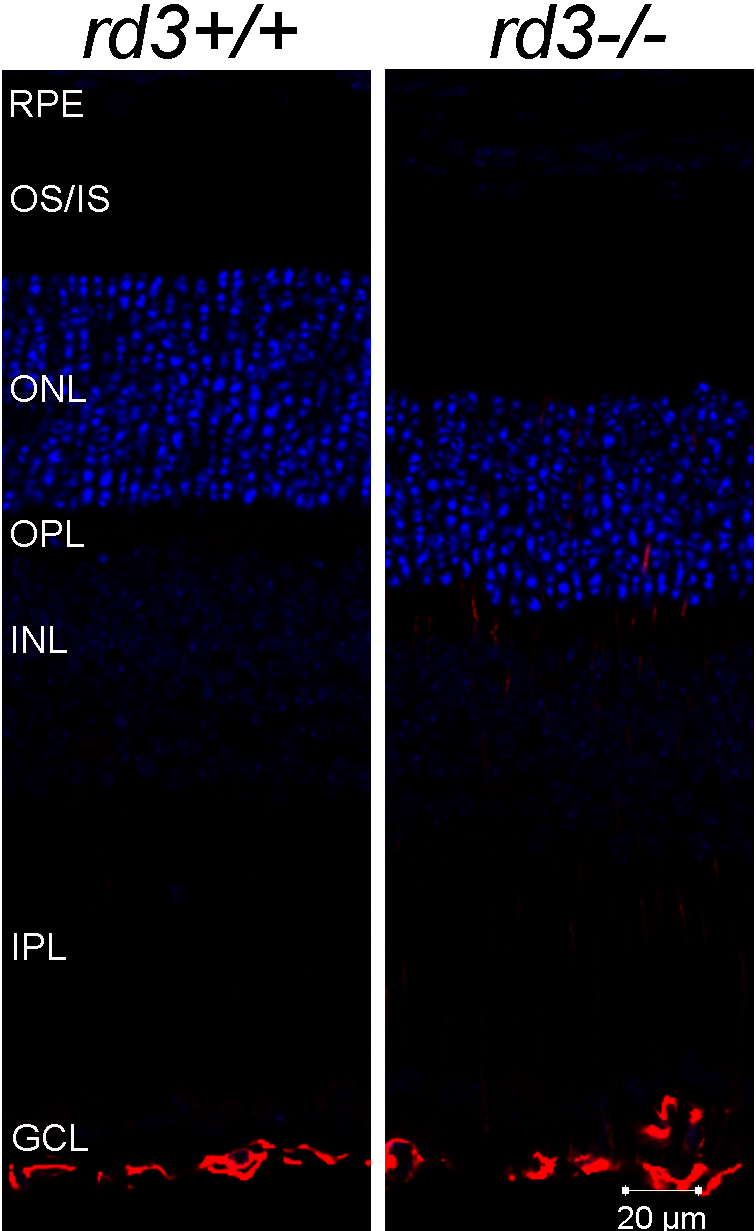
Immunofluorescent labeling of GFAP (red) in retinal cryosections of wild-type (*rd3^+/+^*) and *rd3^−/−^* mouse. Labeling of GFAP is restricted to the Müller cells located at the inner limiting membrane of the retina in the wild-type mouse, whereas in the *rd3* retina, the labeling expanded toward the outer nuclear layer. Abbreviations: RPE, retinal pigmented epithelium; OS/IS, outer segment/inner segment; ONL, outer nuclear layer; OPL, outer plexiform layer; INL, inner nuclear layer; IPL, inner plexiform layer; GCL, ganglion cell layer.

Glial cell activation is another prominent pathway in the *rd3* retina. The marker for glial cell activation, GFAP, is often significantly upregulated in inherited retinal degeneration mouse models and induced retinal injuries. The expression of *Gfap* is elevated by more than 11-fold in the *rd3* retina as measured by the microarray, and the increased expression is confirmed by immunofluorescent labeling of the *rd3* retinal cryosection (Figure 4). In the control retina, anti-GFAP labeling was restricted to Müller cells at the inner limiting membrane (ILM). In contrast, anti-GFAP labeling in Müller cells expanded from the ILM through the inner retina to the outer limiting membrane in the *rd3* retina at P21.

To gain further insight into the biologic meaning of the differentially expressed genes, upregulated genes and downregulated genes were compared using functional annotation tools in DAVID (Table 4, Table 5, Table 6). The method allowed the separate classification of these two gene groups according to the function of the respective protein products (under the organizing principle of Biologic Process and Molecular Function) and the cellular location of these proteins (under the Cellular Component) revealing clear distinctive patterns in the biologic importance of the two groups. Biologic processes associated with the upregulated genes include immune responses, inflammation, apoptosis, vesicle-mediated transport, protein processing, and cellular ion homeostasis (Table 4). The molecular functions facilitating these biologic processes include protein binding, transcription factor activity, chemokine activity, and G-protein-couple receptor binding (Table 5). The cellular components assigned to this group reflect the nature of their biologic processes. A large percentage of this group is categorized as associated with the plasma membrane (16%) where most proteins participating in immune response are expressed. Other cellular components identified in this group are extracellular matrix, cell surface, vacuole, lysosomes, major histocompatibility complex class I protein complex, phagocytic cup, secretory granule, and transcription factor complex (Table 6).

**Table 4:** Comparison of Biologic Processes Associated with the Up-Regulated and Down-Regulated Molecules.

**GO Term: Biologic Processes**	**Count**	**%**	**P-value**
**Upregulated Molecules:**			
Immune system process	55	12.09	2.96E-14
Inflammatory response	29	6.37	6.15E-14
Defense response	39	8.57	4.52E-13
Complement activation, classical pathway	8	1.76	2.71E-06
Cytokine-mediated signaling pathway	10	2.20	2.82E-06
Regulation of programmed cell death	29	6.37	3.29E-05
Regulation of vesicle-mediated transport	10	2.20	7.34E-05
Phagocytosis	8	1.76	8.06E-05
Protein processing	10	2.20	1.38E-04
Regulation of protein kinase cascade	11	2.42	1.98E-03
Intracellular signaling cascade	33	7.25	4.84E-03
Regulation of catalytic activity	20	4.40	5.58E-03
Cation homeostasis	11	2.42	6.71E-03
Regulation of localization	18	3.96	7.21E-03
			
**Downregulated Molecules:**			
Visual perception	10	3.47	1.99E-06
Phototransduction	5	1.74	1.38E-04
Glycerolipid metabolic process	8	2.78	6.35E-04
Eye photoreceptor cell differentiation	4	1.39	2.10E-03
Detection of abiotic stimulus	5	1.74	2.75E-03
Detection of external stimulus	5	1.74	4.62E-03
Phosphoinositide metabolic process	5	1.74	5.50E-03
Phospholipid metabolic process	7	2.43	1.03E-02
Response to radiation	6	2.08	2.28E-02
Actin filament organization	4	1.39	2.51E-02
Glycerolipid biosynthetic process	4	1.39	2.75E-02
Metabolic process	92	31.94	3.35E-02
Neuron development	8	2.78	4.72E-02
Lipid metabolic process	14	4.86	5.05E-02

Separate analysis of upregulated and downregulated genes by the functional annotation program, DAVID, revealed distinct patterns of biologic processes between the two groups of genes. Only a selection of the biologic processes are presented, processes with similar annotation terms are omitted for the interest of conciseness. The number of molecules assigned to each process (Count), the percentage of molecules assigned (%), and the p values are listed.

**Table 5:** Comparison of Molecular Functions Associated with Up-Regulated and Down-Regulated Molecules (DAVID).

**GO Term: Molecular Function**	**Count**	**%**	**P-value**
**Upregulated Molecules:**			
Protein dimerization activity	24	5.27	8.05E-07
Protein binding	162	35.60	6.10E-06
Transcription factor activity	35	7.69	3.71E-05
Chemokine activity	7	1.54	1.25E-04
Transcription regulator activity	44	9.67	3.84E-04
G-protein-coupled receptor binding	8	1.76	6.34E-04
Sequence-specific DNA binding	23	5.05	3.27E-03
Rac guanyl-nucleotide exchange factor activity	3	0.66	4.18E-03
Non-membrane spanning protein tyrosine kinase activity	5	1.10	9.47E-03
Amine transmembrane transporter activity	6	1.32	1.01E-02
Cytokine activity	10	2.20	1.35E-02
Cyclin-dependent protein kinase regulator activity	3	0.66	3.82E-02
			
**Downregulated Molecules:**			
Photoreceptor activity	3	1.04	6.85E-03
Cation binding	53	18.40	3.20E-02
Ion binding	53	18.40	3.96E-02
Metal ion binding	52	18.06	4.03E-02
Phosphoric ester hydrolase activity	8	2.78	4.76E-02
Inositol or phosphatidylinositol kinase activity	3	1.04	5.27E-02

Molecules that are upregulated clearly have different molecular functions than those that are downregulated. The number of molecules assigned to each process (Count), the percentage of molecules assigned (%), and the p values are listed.

**Table 6:** Comparison of Cellular Components Associated with Up-Regulated and Down-Regulated Molecules (DAVID).

**GO Term: Cellular Component**	**Count**	**%**	**P-value**
**Upregulated Molecules:**			
Extracellular region	56	12.31	9.67E-05
Cell surface	17	3.74	5.80E-04
Plasma membrane	76	16.70	6.24E-03
Anchored to membrane	11	2.42	8.34E-03
Vacuole	10	2.20	2.06E-02
Lysosome	9	1.98	2.56E-02
MHC class I protein complex	4	0.88	5.02E-02
Phagocytic cup	2	0.44	5.83E-02
Secretory granule	6	1.32	8.32E-02
Transcription factor complex	9	1.98	9.46E-02
**Downregulated Molecules:**			
Photoreceptor outer segment	3	1.04	2.01E-02
Nonmotile primary cilium	3	1.04	4.05E-02
Sarcoplasmic reticulum	3	1.04	4.52E-02
Intracellular non-membrane-bounded organelle	27	9.38	6.17E-02

Regulation of apoptosis was associated with upregulated transcripts exclusively. There were equal numbers of proapoptotic and antiapoptotic genes according to the gene clustering tool (DAVID). Previous studies on the progress of retinal degeneration identified protective mechanism involving antiapoptotic genes as an early event of degeneration followed by an increase in proapoptotic genes leading to apoptosis in later stages of the disease [5,22]. The implication of the equal number of proapoptotic and antiapoptotic genes observed in the *rd3* retina is consistent with the occurrence of a transition from a protection stage to a deterioration stage.

None of the downregulated genes were classified as involved in apoptosis or immune system processes; instead, these genes are categorized in biologic processes such as phototransduction, the glycerolipid metabolic process, photoreceptor differentiation, and the phospholipid metabolic process (Table 4). The molecular functions involved are photoreceptor activity and ion binding (Table 5). Correspondingly, the cellular components of the protein products of this group include the photoreceptor outer segment and the nonmotile primary cilium (Table 6).

The list of differentially expressed genes identified in the *rd3* retina by this microarray study shares overwhelming similarity with that found in other microarray analyses examining the course of retinal degeneration [5,7,8,22-28]. The fold change and the trend of many genes, especially those involved in immune response and stress response, are highly comparable (Appendix 2), with the exception of *Edn2*, which has a fold increase of 165 in the *rd3* retina, representing an unusually high fold change.

### Confirmation with quantitative real-time polymerase chain reaction

qPCR was used to validate the microarray results for a selection of genes that exhibited increased expression (*Bcl3, C1qa, Edn2, Gdpd3, Gfap*), decreased expression (*Atp8a2, Gucy2f, Mosc1, Pdc, Rgr*), and no change in expression (*Gapdh, Cnga1*; Appendix 3). The expression of *Gucy2e (Gc1)* and *Gucy2f (Gc2)* was also assessed with qPCR because of their relevance to the function of RD3. As shown in Table 7, the trend (increased or decreased) in expression of all genes examined is similar between the microarray results and the qPCR results. The actual fold change varied slightly for some genes, especially with *Edn2*, whose fold change as detected by qPCR is in much more agreement with other microarray studies. Interestingly, *Gucy2e* was not considered significantly differentially expressed by the microarray data because the fold change fell below the 2.0 cutoff; however, the reduced expression as detected by qPCR was more pronounced than that of *Gucy2f*.

**Table 7:** Comparison of Microarray and qPCR Fold Change Data.

**Gene symbol**	**Gene name**	**Microarray** **Fold change**	**qPCR** **Fold change**		
		**P21**	**P14**	**P21**	**P35**
*Atp8a2*	ATPase, aminophospholipid transporter-like, class I, type 8A, member 2	−2.9	+1.2	−3.0	+0.1
*Bcl3*	B-cell leukemia/lymphoma 3	+33.3	+2.0	+7.7	+5.9
*C1qa*	Complement component 1, q subcomponent, alpha polypeptide	+7.6	+2.7	+4.3	+3.8
*Cnga1*	Cyclic nucleotide gated channel alpha 1	−1.3	+0.3	−0.4	−0.2
*Edn2*	Endothelin 2	+165.2	+2.1	+9.4	+5.2
*Gdpd3*	Glycerophosphodiester phosphodiesterase domain containing 3	+16.4	+1.3	+7.3	+1.7
*Gfap*	Glial fibrillary acidic protein	+11.4	+1.6	+3.9	+4.1
*Gucy2e*	Guanylate cyclase 2e	−1.9	+0.8	−4.1	−0.3
*Gucy2f*	Guanylate cyclase 2f	−3.0	+0.4	−2.9	−0.4
*Mosc1*	MOCO sulphurase C-terminal domain containing 1	−8.4	−3.3	−3.7	−3.6
*Pdc*	Phosducin	−4.7	−0.1	−3.1	−0.4
*Rgr*	Retinal G protein coupled receptor	−4.2	−0.2	−5.2	−0.2

Positive values indicate an increase in expression, and negative values indicate a decrease in expression. Three mice were used per time point in qPCR experiment.

The expression level of the selected genes was examined at additional time points (P14 and P35) to get a sense of the rate of expression changes (Table 7). At an earlier time point (P14), most genes had similar expression compared to the wild-type, except *Mosc1*, which had a threefold decrease. At P35, some of the selected genes had returned to the wild-type level while other genes (*Bcl3*, *C1qa*, *Edn2*, *Gfap*, and *Mosc1*) retained their differential expression level as seen at P21, suggesting their importance in the progression of photoreceptor degeneration in the *rd3* mice.

## Discussion

Mutations in the *RD3* gene are associated with LCA12 [11,12]. LCA12 is a devastating disease causing blindness at birth or severe visual impairment before the first birthday. In mice, a mutation in the *Rd3* gene also causes loss in vision albeit more gradually depending on the genetic background of the mouse strain. The mouse *Rd3* gene is preferentially expressed in the retina and displays an increased level of expression during early postnatal development [11]. Recent studies by Azadi et al. [16] and Peshenko et al. [17] showed that RD3 is a photoreceptor guanylate cyclase-binding protein and this interaction contributes to the stable expression of GCs and regulation of their enzymatic activity. However, the pathogenic mechanisms underlying photoreceptor degeneration in the *rd3* mouse remain unsolved. The current study explores this question by analyzing the global genomic changes in the *rd3* retina to identify genes and molecular pathways that are potentially involved in photoreceptor degeneration.

### Potential pathways affected in *rd3* retina

The separate analysis of the upregulated and downregulated genes with DAVID revealed a striking distinction in the biologic processes between these two categories of differentially expressed genes (Table 4). The upregulated genes are involved in immune responses and apoptosis, processes that may contribute directly to the demise of photoreceptors. In contrast, the downregulated genes are involved in physiologic processes such as phototransduction and lipid metabolism, possibly resulting from the lack of a functional RD3 protein.

### Immunological responses

The microarray analysis of the *rd3* retina revealed significant changes in the expression of an overlapping set of immune response genes found in several models of retinal degeneration and human retinal diseases. Accordingly, the immunological pathways identified by IPA from this set of genes (Table 3) have also been observed in previous studies. In the human glaucomatous retina, tumor necrosis factor receptor 1 signaling that triggers apoptosis has been documented [29]; whereas acute phase response, an early response to tissue injury, infection, or inflammation, has been reported in diabetic rats [30]. Endothelin 1 signaling is believed to be important in regulating retinal blood flow [31] during an immune response, and is upregulated in mouse and rat models of glaucoma [32,33]. Complement cascade is a reoccurring theme in naturally and induced-degenerating retina [22,24,32,34] because it is the major means by which the body clears pathogens and promotes inflammation. This striking similarity has led to the suggestion that similar immunological responses as listed above are activated upon the initial stage of photoreceptor degeneration in various retinal disease models regardless the origin of genetic mutation. Our microarray study shows that *rd3*-induced photoreceptor degeneration in the 4Bnr-BALB/c-*Rd3^rd3/rd3^* mice model follows parallel pathological mechanisms.

Glial activation is another key event that has been observed in models of various inherited photoreceptor degenerations [3,8,25], a genetically engineered model (*Nrl*-knockout [26], a light-damaged model [8,35], and other stress-induced retinal injury models [4,5], as well as non-ocular central nervous system disease and injuries [36]. Activation of glial cells in the retina can be neuroprotective through the release of neurotrophic factors such as basic fibroblast growth factor (bFGF) and ciliary neurotrophic factor (CNTF) [37] or antioxidants such as glutathione [38]. At the same time, glial cells can also cause damage to retinal tissue by dedifferentiating in response to intense injury resulting in impaired neurotransmitter removal and homeostasis of ion and water [39].

The detailed mechanism of glial cell activation in the context of retinal degeneration was explored in a recent study [40] that investigated the gene expression changes taking place in Müller glial cells at the peak of photoreceptor cell death. GFAP and vimentin are markers of gliosis and were highly upregulated in that study. Similarly, in our study the expression of these two genes was increased by more than 11- and twofold, respectively, and the elevated expression of GFAP was sustained even at P35 as quantified with qPCR. Proteins involved in detoxification that were coregulated with GFAP were metallothionein (MT), an antioxidant toxic metal binding protein, and ceruloplasmin (CP), a copper binding ferroxidase involved in iron homeostasis, both of which were increased by more than fivefold in our *rd3* study. An immune response–related gene, *Cebpd*, that regulates macrophage activation was upregulated in Müller cells and by more than 16-fold in our study.

### Phototransduction pathway

The expression of most photoreceptor-specific genes, such as *Rho*, *Abca4*, *Cnga1*, or *Pde6*, was not significantly decreased as might be expected for a retina with such obvious signs of photoreceptor loss. However, the photoreceptor-specific genes found differentially expressed in the *rd3* retina all participate in the phototransduction pathway. These are *Opn1sw, Gnat1*, *Pdc*, *Guca1b,* and *Gucy2f*, all of which were significantly downregulated, and *Gnb3*, which was upregulated. The distribution and expression of *Opn1sw* (blue opsin), together with red/green opsin, were downregulated in the GC1 single and GC1/GC2 double knockout mouse [41]. Dramatic reduction in blue opsin expression has also been demonstrated in a few other retinal degeneration models, including *Bbs4*-knockout mice [24].

*Gnat1* and *Gnb3* encode the alpha (Tα) and beta (Tβ) subunits of the guanine nucleotide regulatory protein, transducin. These two subunits together with Tγ form a heterotrimeric complex that binds to rhodopsin and converts the light stimulus detected by rhodopsin into a chemical signal by activating the hydrolysis of cGMP by phosphodiesterase (PDE). The activity of transducin is regulated by phosducin (PDC) through the sequestering of the Tβ/γ subunits to prevent the reassociation with the Tα subunits.

*Gucy2f* (*Gc2*) encodes an enzyme that catalyzes the synthesis of cGMP from GTP, thus replenishing the cGMP broken down by PDE. The activity of GC is regulated by the calcium-sensing GCAP. *Gcap2 (Guca1b)* was identified as one of the qualitative trait loci (QTL) located on chromosome 17 that modulates the *rd3* disease [42]. Though a mutation in *GCAP2* has been linked to autosomal dominant retinitis pigmentosa in a group of Japanese patients [43], the mutation has not been linked to other populations, unlike *GCAP1* [44]. Similarly, no human retinal disease has been linked to *GC2* yet, and the mouse retina lacking GC2 has a normal ERG [41], suggesting that GC1 alone is sufficient to compensate. The decrease in expression of *Gc1* and *Gc2* as detected by a combination of microarray analysis and qPCR experiments suggests that RD3 may regulate the expression and stability of GCs at the transcriptional level, and further attest to the functional significance of RD3 in phototransduction signaling.

### Lipid metabolism

Lipids have numerous profound biologic roles including membrane biosynthesis and stability, the regulation of endocytosis and phagocytosis, the activation of inflammation, and signaling. Of particular interest to the *rd3* retina are the membrane structure and signaling aspects of lipid metabolism. First, the genes involved in membrane integrity that were differentially expressed in the *rd3* retina are *Pnpla3* and *Pnpla8*, which are calcium-independent phospholipases that cleave membrane phospholipids; *Pla2r1*, a receptor for phospholipase; *Pla1a*, which hydrolyzes phosphatidylserine; and *Pla2g7*, an enzyme that hydrolyzes oxidized phospholipids generated during normal light exposure. *Pla2g7* is another QTL that influences photoreceptor degeneration in *rd3* mice [42], and downregulation has been detected in other mouse models including *rd1*, *rd2*, and constant-light damage in BALB/c [7]. Second, genes affecting the asymmetry of the plasma membrane that were differentially expressed in the *rd3* retina are the aminophospholipid transporters, ATP8A2 and ATP9B. ATP8A2 has been implicated in generating and maintaining phosphatidylserine asymmetry in photoreceptor disc membranes of rods and cones [45].

**Figure 5:**
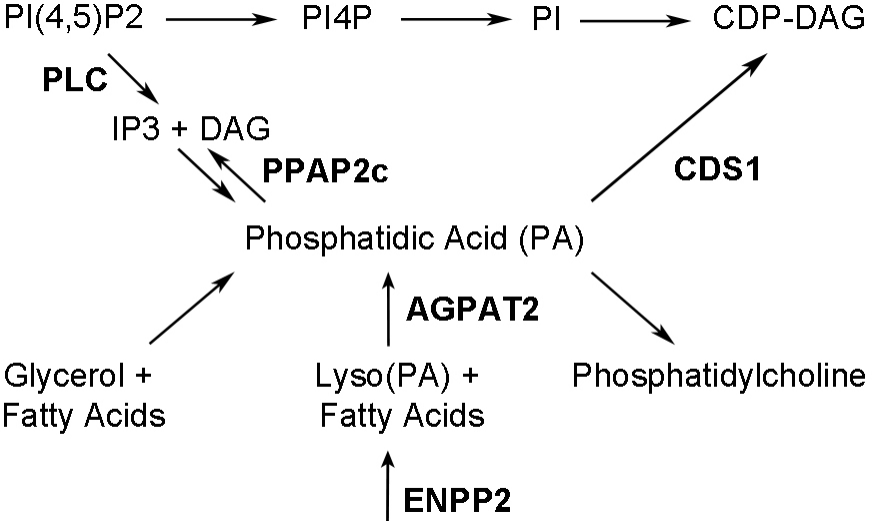
Lipid metabolism involving phosphatidic acid (PA). The expression of a few key enzymes (shown in the figure) involved in the direct metabolism of PA are affected in the *rd3* retina. CDS1 (CDP-diacylglycerol (DAG) synthase) is an enzyme that condenses PA with CTP to produce CDP-DAG, an essential step in generating phosphatidylinositol (PI). The lipid biosynthetic enzyme AGPAT2 (1-acyl-sn-glycerol 3-phosphate O-acyltransferase 2) converts lyso(PA) into PA, which is needed to produce lipids important for cell membranes and chemical signaling within cells. Lyso(PA) is generated by ENPP2 (ectonucleotide pyrophosphatase/phosphodiesterase 2). PPAP2c (phosphatidic acid phosphohydrolase type 2c) catalyzes the conversion of PA to DAG. Figure is adapted from Raghu et al. [46].

Finally, the genes involved in lipid signaling seem to converge onto the phosphatidic acid (PA) pathway (Figure 5). As a major source for phototransduction cascade and photoreceptor membrane, PA is an intermediate for generating second messengers such as IP3 and DAG, PI and PG, as well as glycerol and fatty acids. Abnormal levels of PA-metabolizing enzymes would result in disruption of membrane structure and signaling within the photoreceptors. In Drosophila, elevated levels of PA have been found to perturb membrane transport to the apical domain in photoreceptors [46]. It is tempting to speculate that RD3 plays a central role in the metabolism of PA as the expression of many enzymes directly involved in this pathway are mis-regulated in the *rd3* retina.

### Other potential pathogenetic mechanisms in retinal degeneration

In addition to the overt involvement of immune response observed in various models of retinal degeneration, defects in phototransduction, ion transport mechanisms, and signaling seem to be characteristic of the rod photoreceptor death. Dysregulation of genes involved in these pathways have been reported in the *rd1* mouse at the peak of rod death (P14) [3]. Disruption in lipid signaling and phototransduction has been observed in the *rd3* retina. Abnormal expression of numerous genes involved in ion transport was also noted in the microarray. Genes including solute carrier organic anion transporter (*Slco1c1*), solute carrier family 5 member 2 (*Slc5a2*), and solute carrier family 24 member 1 (*Slc24a1*) were downregulated by more than three-, ten-, and twofold, respectively, in the *rd3* retina. The mRNA of *Slco1c1* has been detected in the retinal pigmented epithelium and the inner nuclear layer of the retina and has been implicated in organic anions transport in ocular tissue [47]. SLC5A2 is a sodium-dependent glucose transporter and may be involved in diabetic retinal cell death [48]. Mutations in *SLC24A1*, a gene encoding the photoreceptor-specific sodium/calcium exchanger, have been linked to autosomal recessive congenital stationary night blindness [49].

Mitochondrial oxidative stress preceding degeneration and ER stress coinciding with photoreceptor death have been demonstrated in *rd1*, *Rds*, and *Rho^−/−^* mice [50,51]. Our microarray data did not detect the activation of such stress responses perhaps due to our later time point of analysis, but these events are of potential interest for understanding the entire course of photoreceptor degeneration.

### Conclusion

RD3 is an interacting partner of GCs, and this interaction is crucial for the stable expression of the GCs in photoreceptor cells. Consistent with this, GCs are not detectable in the *rd3* retina. *Rd3* mice exhibit many similarities to GC double knockout mice in that they demonstrate a loss of GCs and GCAPs, shortened photoreceptor outer segments, and progressive photoreceptor degeneration. However, differences exist between the two models. For example, the onset and rate of photoreceptor degeneration are more aggressive in the *rd3* retina than in the GC double knockout, suggesting RD3 plays a broader role in photoreceptor physiology. Pathogenic mechanisms outside the context of GC inactivity, namely, the loss of cGMP production and the subsequent prolonged reduction in the intracellular calcium level, were envisioned for the *rd3* retina. This microarray study identified aberrant expression of genes involved in phototransduction and lipid metabolism, implicating defects in these pathways could be the underlying causes of the photoreceptor degeneration induced by the mutation in the *rd3* mouse. Future studies examining different time points and the protein levels of the gene candidates will shed further light on the pathogenesis of LCA12.

## Appendix 1.

Gene expression changes in the *rd3* retina at P21 as determined by Agilent SurePrint G3 microarray technology. Fold changes are calculated using normalized values, which are the absolute value of the ratio of the *rd3* averages over the wild-type averages. Differentially expressed genes with a fold change greater than 2.0 and a p value cutoff of 0.05 are reported. To access the data, click or select the words “Appendix 1.”

## Appendix 2.

A Comparative Look at the Fold Change of Gene Expression at the Peak of Photoreceptor Death between Models of Retinal Degeneration. The table presents some of the genes and pathways often observed in microarray studies on various models of photoreceptor degeneration, including naturally-occurring mutations, genetically-engineered, and injury-induced models. The selected microarray studies is by no mean an exhaustive list. (*rd3* (P21), our study; *rd1* (P10) [7]; *rd2* (P21) [7]; *Rds* (P21) [23]; *Bbs4^−/−^* (5 mo) [24]; *Rs1h^-/y^* (P14) [25]; *prCAD^−/−^* (1 mo) [8]; *Nrl^−/−^* (4 mo) [26]; *Nxnl1^−/−^* (P40) [27]; Light-damaged (LD, 24 h) [7,8]; Retinal detachment (24 h) [22,28]; Hyperoxia [5]). To access the data, click or select the words “Appendix 2.”

## Appendix 3.

A List of PrimeTime qPCR Assays. A selection of genes were chosen for the qPCR study to validate the microarray results. Each PrimeTime qPCR assay includes a pair of primers and a probe for the target gene. *Gapdh* was used as a reference gene for normalization. To access the data, click or select the words “Appendix 3.”
